# Emerging Forms of Precariousness Related to Autonomy at Work: Toward an Empirical Typology

**DOI:** 10.3389/fsoc.2020.00034

**Published:** 2020-05-22

**Authors:** Louis Florin, François Pichault

**Affiliations:** HEC Management School, University of Liège, Liège, Belgium

**Keywords:** autonomy, dependence, self-employed, typology, non-standard work arrangements, Ipros, cluster analysis

## Abstract

Societal, technological, and economical changes in the last decades have led to the development of new work arrangements located in a ≪ gray zone ≫ between standard employment and classical self-employment (Cappelli and Keller, 2013a; ILO, 2016; Katz and Krueger, 2016). Official labor market statistics must be adapted to provide researchers and policymakers with relevant data on this population (Gazier et al., 2016; National Academies of Sciences, Engineering and Medicine, 2017; ILO, 2018). Cappelli and Keller (2013b) point out that new work arrangements are characterized by changes in the management of the work relationships (with a growing intervention of labor market intermediaries) and in the way the work is supervised (from work processes to outcomes). The concept of autonomy thus becomes a central feature of new work arrangements leading to specific configurations of risks and opportunities for individual workers concerned. Autonomy can be divided in three main dimensions: work status, work content, and working conditions (Pichault and McKeown, 2019). International surveys such as the European Working Conditions Survey (EWCS) provide valuable data covering these dimensions of autonomy. Our paper is focused on a specific category of workers experiencing the ambiguities of autonomy at work: Independent Professionals (Ipros). Ipros provide various forms of intellectual work in the service sector through self-employment and are often regarded as a highly autonomous workforce (Leighton and Brown, 2014; McKeown, 2015) while they can also be subject to precarious situations regarding their economic dependency or freedom of choice (de Peuter, 2011; Standing, 2011; Bergvall-Kåreborn and Howcroft, 2013). The objectives of this paper are, first, to build a set of indicators likely to measure the various dimensions of autonomy, and, second, to provide an empirical typology of new work arrangements by using cluster analysis methods. Through the application of this analytical framework on the EWCS 2015 data, we observe various situations in terms of risk and opportunities related to autonomy, shedding light on unexpected precarious situations where Ipros face the risks of autonomy without getting the associated benefits. Our results provide a nuanced typology of empirical situations, overcoming such a dichotomic vision of non-standard work arrangements.

## Introduction

Societal, technological, and economical changes in the last decades led to the development of new employment arrangements that sits in a ≪ gray zone ≫ between classical statuses of self-employment and salaried work (Cappelli and Keller, 2013a; Eurofound, 2015; ILO, 2016; Katz and Krueger, 2016). As the need for insightful data on this population is growing, official labor market statistics still must be adapted to allow researchers and policymakers to catch the phenomenon (Gazier et al., 2016; National Academies of Sciences, Engineering and Medicine, 2017). The objectives of this paper are, first, to develop and test the validity of indicators of autonomy based on the European Working Conditions Survey 2015 and, second, to provide an empirical typology of employment arrangements by using cluster analysis methods.

## Background

### Official Statistics Typologies: the Classical Approach

New forms of employment are commonly reported as employment arrangements that differ from the traditional open-ended salaried contract: fixed-term contract, part-time work, and self-employment (Everaere, 2014; Schmid, 2015; ILO, 2016). This classical approach allows statisticians produce regional or international comparisons, but fails to make visible the diversity of new forms of employment. Indeed, fixed-term contract, part-time work, and self-employment are still reported as new forms of employment even though they have represented a fair share of the working arrangements for a long time. They do not help understand emerging forms of employment. Moreover, there is a wide variety of employment arrangements that fit in the same working status. Under the self-employed status for example, we find arrangements going from economically dependent one-client subcontracting to multi-client and completely autonomous independent contracting or intermediated work relations. This approach thus fails in capturing the gray zone of working arrangements that share characteristics of both traditional statuses: self-employed and salaried work. Gazier et al. (2016) pointed out that the typology of employment arrangements in official statistics should be reviewed and that more relevant information should be produced, among others, on intermediated forms of employment (co-employment, subcontracting) and freedom of choice for contingent work. Cieslik (2015) showed that administrative business registers lack important information for understanding contemporary self-employment. Other international organizations and researchers pointed out the shortcomings of the existing statistical data and developed new classifications. We can identify *ad-hoc* and generic approaches.

### *Ad-hoc* Classifications

Some researchers have developed *ad-hoc* definitions to fit specific forms of employment such as the Independent Professionals, Interim management, Portfolio work, On-call workers, and so on (Eurofound, 2015; Katz and Krueger, 2016). These researches shed light on some specific parts of the workforce and provide a more refined and valuable insight to researchers and policymakers. However, many of the concepts used in these studies are not yet stabilized in the scientific community and are very dependent on the type of data used. This lack of international uniformization of definitions and categories between international organizations or researchers leads to a wide variety of listings of new forms of employment that brings some confusion. The lack of shared definitions and concepts and the non-exclusivity between categories usually prevent such methods to be generalized.

### Generic Typologies

Some approaches take a more general perspective. Cappelli and Keller (2013b) suggest a typology of working arrangements that relies on the type of authority and control that the employer/client has over the worker. Their classification first distinguishes employment (where control is focused on the work process) and contract work (where control is focused on the outcomes) and secondly looks at the potential intervention of a third party to distinguish co-employment from direct employment or again direct contracting from subcontracting. New work arrangements are characterized by more control on the outcomes and shared supervision between different parties, sometimes becoming evanescent. In these conditions, autonomy at work becomes a central feature in many modern work arrangements. This notion will be at the core of our analysis and will be developed further in the paper.

Authority, autonomy and dependency have also played a role in rethinking international classifications of employment arrangements. The scientific and political debates around new forms of employment and their classification have led the 20th International Conference of Labor Statisticians organized by the International Labor Office (ILO) to review the International Classification of Professional Situation adopted in 1993 (CISP-93). This classification is still the international reference for official statistics and international surveys. To respond to the increasing demand of relevant data on emerging work arrangements, a new classification has been adopted at the conference (ILO, 2018). This new classification (CISE-18) will consider the type of authority and the economic risk faced by workers to create new categories, such as the ≪ non-salaried dependents ≫. It also aims at shedding light on multiparty work relations. While this is certainly an important step for labor statisticians and decision-makers, the implementation of such new classification in official statistics and international surveys should unfortunately take some time.

### Surveys and Empirical Typologies

For Desrosières (2005), as administrative data are made by the state to be able to manage, they better reflect the way the institutions work while surveys allow to explore society more specifically according to the needs of statisticians. International surveys such as the Labor Force Survey from Eurostat, the European Social Survey and the European Working Conditions Survey from Eurofound gather in-depth data about the labor situation of workers. By adding questions about quality of work, working conditions, vulnerability, autonomy, and risks, these surveys provide information that goes beyond work statuses. There has been a lot of work to develop indicators of job quality (Eurofound, 2012), or job vulnerability (Bazillier et al., 2016) based on these surveys. Since 2012, the indices of job quality developed by Eurofound have been included in many reports. They measure earnings, job prospect, intrinsic job quality (skills and discretion, social environment, physical environment, and work intensity) and working time quality.

For the 6th wave of the EWCS, following the debates regarding new forms of employment, Eurofound extended the number of questions asked to self-employed workers, by adding questions regarding their working situation, their economic dependency or their income (Eurofound, 2017a). Some recent work extended the job quality approach to all statuses (Eurofound, 2018a), showing that dependent and independent solo self-employed workers experience lower scores on employment prospects, skills and discretion, physical and social environment, and work intensity while self-employed workers with employees have a relatively high job quality.

This approach by indices has led to a new form of classification. To go further and look beyond statuses and/or theoretical classifications, some researchers tried to develop an empirical approach to classifying workers. Such empirical typologies are less based on predetermined conceptual definitions and more related to the scores resulting from various dimensions and indicators. As workers belonging to the same statistical category can have very different experiences in terms of employment arrangements, empirical classifications take a bottom-up approach that groups workers sharing similar scores on several dimensions together. These classifications use cluster analysis methods.

Cluster analyses based on job characteristics of salaried workers provide interesting typologies that show which categories of workers are at risk. The first cluster analysis performed by Eurofound on job quality indices identifies four clusters: high-paid good jobs, well-balanced good jobs, poorly balanced jobs, and low-quality jobs (Eurofound, 2012). Van Aerden et al. (2014) developed other measures of employment quality based on EWCS (employment instability, material rewards, worker's rights and social protection, working time arrangements, employability opportunities, collective organization, and power relations) in order to provide a typology of employment arrangements. Their aim is to show how various employment relationships differ from standard employment by postulating that de-standardization of employment is not only a matter of status but requires a multidimensional approach. Their classification identifies five clusters: Standard Employment Relationship-like jobs, instrumental jobs, precarious unsustainable jobs, precarious intensive jobs, and portfolio jobs. In Belgium, Vandenbrande et al. (2012) identified 22 sub-dimensions of job quality and conducted a cluster analysis that produced seven categories: saturated jobs, full-time balanced work, work with limited career prospects, work on flexible and unusual hours, emotionally demanding job, heavy repetitive work, and indecent work.

While these studies revealed the variety of employment situations and the de-standardization processes of salaried work, we still lack information about self-employed workers. Recently, a deeper focus on self-employment has been provided by Eurofound for the 6th EWCS 2015. Researchers have developed new classifications of self-employment using the self-perceived status, the magnitude of economic activity and the economic dependency (Eurofound, 2017b). However, as the self-perceived status is highly dependent on national contexts, they also developed an empirical classification of self-employed workers. Building on such variables as entrepreneurialism, economic and operational dependency and economic sustainability/precariousness, the analysis classifies self-employed workers in five clusters: employers, small traders and farmers, stable own-account workers, vulnerable workers, and concealed workers (Eurofound, 2017c). This approach allows policymakers and researchers identify which categories of self-employed workers are at risk. However, it seems that this classification reproduces existing categories (employers vs. solo) or sectors (farmers and traders) and therefore prevents identifying the main characteristics of new employment arrangements.

### Toward an Empirical Classification of Independent Professionals Based on Multiple Dimensions of Autonomy

This has led to precious insights on the diversity of self-employment situations. Yet, new forms of employment are characterized by significant changes in subordination links and in the way the work is supervised (Cappelli and Keller, 2013b). As shown in several empirical studies devoted to new forms of employment, the most relevant changes in this kind of jobs can be characterized by the concept of autonomy [Leighton and McKeown (2015); Bush and Balven (in press)]. According to the conceptual matrix provided by Pichault and McKeown (2019), autonomy can be divided in three main subdimensions: work status (how the access to social protection is guaranteed), work content (which kinds of work division and coordination mechanisms are provided), and working conditions (who is responsible for skills development, income generation, time and space arrangements).

Table 1 represents these different dimensions of autonomy. Regarding *work status*, we can notice various situations that fit in between employed and self-employed work, such as co-employment and work supported by third parties (like platforms). These options can be mixed with diverse modalities in terms of social protection, number of business partners, economic dependency and freedom of choice. The *work content* may be based on broad guidelines and low control which paves the way to job crafting, full responsibility regarding the working pace and load, flexible coordination mechanisms and strong support from the professional community against managerial intrusions. But the work content can also be based on tight controls, with few possibilities of job crafting, imposed working pace and load, rigid coordination mechanisms and no access to professional support against managerial intrusions. In terms of *working conditions*, the responsibility for skills development, income generation and space and time arrangements can be entirely left to the worker, facilitated by third-party organizations, negotiated with or imposed by the client. It is assumed that all these dimensions can vary independently from each other.

**Table 1:** Autonomy at work of Independent Professionals (from Pichault and McKeown, 2019).

**High autonomy** 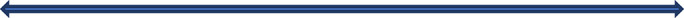 **Low autonomy**			
**WORK STATUS**			
Independent contractor	Supported independent contractor	Temporary worker	Regular employee
Private insurance	Insurance packages via third parties	Discontinuous access to social rights	Continuous access to social rights
Diversity of clients		Economic dependency/sole client	
Deliberate choice		Forced choice	
**WORK CONTENT**			
Broad guidelines allowing job crafting		Detailed specifications preventing job crafting	
Work pace, workload at own discretion		Work pace, workload imposed by clients	
Mutual adjustment Standardization of norms	Standardization of outcomes		Standardization of work processes Direct supervision
Strong support and/or access to shared expertise and practices, high identification to a professional community		Few support and/or access to shared expertise and practices, low identification to a professional community	
**WORKING CONDITIONS**			
Self-responsibility for developing skills	Access to functional equivalents for skills development	Customized skills development plans based on *ad hoc* negotiations	Standardized training policies
Self-responsibility for steady income flow	Financial support offered by third parties	Individualized salary packages from interpersonal negotiations	Standardized salary grids
Self-responsibility for time and space arrangements	Access to shared facilities (co-working)	*Ad hoc* time and space arrangements resulting from interpersonal negotiations	Predetermined work schedules and space arrangements
**High autonomy** 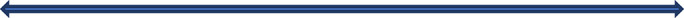 **Low autonomy**			

In order to avoid an implicit reproduction of sector-based and/or job-based distinctions in our typology, such as in the Eurofound (2017c) study, we will focus our analysis on one single group of non-standard workers, supposedly more homogeneous: “independent professionals” (Ipros). Ipros provide various forms of intellectual work in the service sector through self-employment. The term Ipros covers activities such as copywriting, translating, IT, marketing, consulting, creative activities, etc. They are acknowledged as the fastest growing sector in the Western economies workforce. Over the last decade, they have been growing by 45% in the EU (Eurofound, 2015).

IPros are often presented as workers having deliberately chosen the self-employed status (Leighton and Brown, 2014). According to some surveys, they are motivated by autonomy, independence and choice in their work (Leighton and Brown, 2014; McKeown, 2015). The intellectual nature of their job, as opposed to manual work, is usually seen as allowing workers to enjoy higher levels of autonomy (Sandberg and Pinnington, 2009). It seems that traditional bureaucratic control is not easily applicable to such intellectual tasks (Thompson et al., 2009; Wynn, 2016). Other researchers however question this taken-for-granted association between intellectual work and autonomy. IPros do not always individually choose to work as self-employed. Their status sometimes results from constrained choices and might lead to precarious situations and economic dependency (de Peuter, 2011; Standing, 2011; Bergvall-Kåreborn and Howcroft, 2013). Such contrasted results in the literature suggest a more nuanced approach in analyzing their work arrangements.

In this paper, we will build a series of indicators of autonomy according the various dimensions of Table 1, by referring to the 6th European Working Conditions Survey (2015); we will test their validity on the population of IPros. We will then use cluster analysis methods to provide an empirical typology of employment arrangements among Ipros, based on the multiple dimensions of autonomy at work.

## Data and Methods

### Data

This study is based on a secondary analysis of publicly available data (Eurofound, 2018b). EWCS is one of the most comprehensive survey regarding autonomy and its subdimensions. To narrow down our analysis on the independent professionals, we used the operational definition of Ipros by Rapelli (2012): “Self-employed workers, without employees, which are engaged in an activity which does not belong to the farming, craft or retail sectors. They engage in activities of an intellectual nature and/or which come under service sectors.” We therefore selected self-employed workers without employees in the following NACE^1^ codes: Information and communication (J), Financial and insurance activity (K), Real estate activities (L), Professional, scientific and technical activities (M), Administrative and support services (N), Education (P), Human health and social work (Q), Arts, entertainment and recreation (R), and Other service activities (S).

In the 6th wave of the EWCS (Eurofound, 2018b), the sample of IPros consists of 1,345 workers in Europe. We used the weighting variable from the EWCS to control for survey design, post-stratification and supranational weights.

### Methods

This methodological choice means that we were limited to a secondary analysis of existing data, not gathered in our conceptual perspective. This unavoidably led us to some redefinitions of our initial ambitions.

For each sub-dimension of the conceptual grid of autonomy presented in Table 1, we looked for specific questions that can provide us with the appropriate information to develop proxy indicators. However, the EWCS survey did not provide us with relevant questions regarding two dimensions presented in the conceptual grid. Regarding the work status dimensions, there is no question related to social rights and insurances. Regarding the work content, we were able to build proxy indicators for each dimension of the grid. For the working conditions, we could not develop an indicator for skills development as the questions regarding training are only quantitative (number of days spent in training) but do not inform us about the responsibility for training (is the worker the sole responsible for his/her skills development or do the client provide possibilities for training?). This was also the case with the responsibility for spatial arrangements. We were therefore condemned to refer to one single dimension (the management of working time) to build our indicator. Moreover, due to the lack of information about intermediated work relationship, we were not able to find information about some of the possibilities developed in the conceptual grid such as supported independent contracting or financial support offered by a third-party. Table 2 synthetizes the questions and information used in the construction of each indicator.

**Table 2:** Summary of the work autonomy sub-dimensions indicators.

**Indicators**	**Questions in EWCS 2015**	**Information used**
Independence in the contractual arrangement (short: Contract)	Q8b	Q8b. Select the category or categories which apply to your main paid job?—Sole director of own business—A partner in a business or professional practice—Working for yourself—Working as a sub-contractor—Doing freelance work—Paid a salary or a wage by an agency
Economic independency (short: Econ. independency)	Q9d, Q102	Q9d. Regarding your business, do you generally, have more than one client or customer?—Yes—No/Q102—What proportion of revenue do you receive from your most important client?—<50%-−50 to 75%—More than 75%
Choice for self-employed work (short: Choice)	Q10	Q10—Self-employed, was it mainly your own personal preference or you had no better alternatives for work?—Mainly through own personal preferences—No other alternatives for work—A combination of both
Autonomy in work methods (short: Work Methods)	Q54b, Q61i, Q61n	Q54b. Are you able to choose or change your methods of work—Yes—No/Q61i—You are able to apply your own ideas in your work?—Always—Most of the time—Sometimes—Rarely—Never/Q61n—You can influence decisions that are important for your work?—Always—Most of the time—Sometimes—Rarely—Never
Autonomy in work pace (short: Work Pace)	Q54c	Q54b. Are you able to choose or change your pace of work—Yes—No
Coordination mechanisms (short: Coord. Mech)	Q50abcde	Q50acde. On the whole, is your pace of work dependent on—the work done by colleagues—direct demands from people such as customers, passengers, pupils, patients, etc.—numerical production targets or performance targets—automatic speed of a machine or movement of a product—the direct control of your boss
Support/Access to shared expertise (short: Support)	Q58, Q61a	Q58. Do you work in a group or team that has common tasks and can plan its work?—Yes—No/Q61a Your colleagues help and support you—Always—Most of the time—Sometimes—Rarely—Never
Responsibility for generating income (short: Earnings responsibility)	Q103abc	Q103. What do your earnings from your main business include?—Income from self-employment such as own business, profession or farm—Payments based on the overall performance of the company (profit sharing scheme) or partnership where you work—Income from shares in the company you work for
Autonomy in time arrangements (short: Worktime)	Q42	Q42. How are your working time arrangements set?—They are set by the company/organization with no possibility for changes—You can choose between several fixed working schedules determined by the company/organization—You can adapt your working hours within certain limits (e.g., flextime)—Your working hours are entirely determined by yourself

We then aggregated these questions to build synthetic indicators using a normalized scale from 0 (less autonomy) to 1 (more autonomy) for each sub-dimension^2^. We controlled the indicators by reviewing their distribution and descriptive statistics in order to avoid aberrant results.

First, we used univariate analyses of key dimensions to highlight the variety of Ipros' experience of autonomy (section Ipros' Experiences of Autonomy) and we tested the potential correlations between these dimensions (section Autonomy as a Multidimensional Concept). Second, we provided an empirical typology of new work arrangements by using cluster analysis methods (section Building an Empirical Typology).

## Findings

### Ipros' Experiences of Autonomy

**Figure 1:**
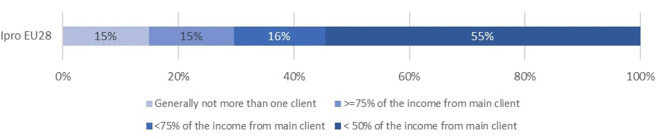
Economic independency.

**Figure 2:**
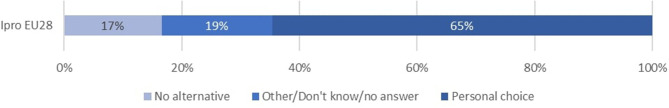
Choice for self-employed work.

**Figure 3:**
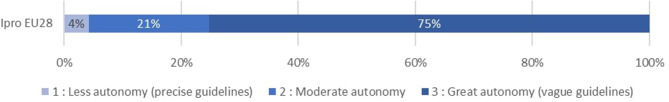
Autonomy in work methods.

**Figure 4:**
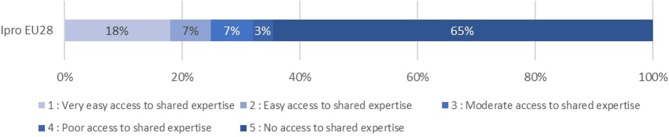
Support/access to shared expertise.

**Figure 5:**
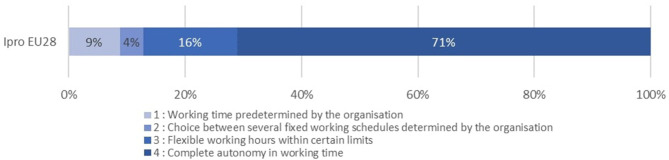
Autonomy in time arrangements.

To understand the experience of autonomy by Ipros through the various dimensions of our matrix, we looked at distributions after having split continuous variables in classes to facilitate visualization and interpretation^3^. We select five dimensions that depict the high variety of I-Pros' experiences of autonomy^4^. Figure 1 denotes the strong proportion of IPros in a situation of economic dependency (30%). Figure 2 demonstrates that at least 17% of IPros work as self-employed because they have no alternative. Figure 3 reveals that 25% of these workers have a low to moderate autonomy regarding the way they execute their tasks while, Figure 4 shows that the majority of Ipros have a limited access to support from colleagues and/or managers. Figure 5 points out that 29% of them are submitted to some kind of external control over their working time arrangements.

These results indicate that the Ipros' experiences of autonomy are diversified. While most of them seem to enjoy high levels of autonomy, there is a non-negligible part experiencing lower levels of autonomy on some dimensions. The second part of our analysis questions the relations between these dimensions.

### Autonomy as a Multidimensional Concept

We then decided to test empirically whether the various sub-dimensions of the matrix can vary independently from each other. We conducted bilateral correlation analyses on these 9 sub-dimensions. Table 3 displays the correlation matrix.

**Table 3:** Indicators correlation matrix.

	**Contract**	**Econ. independency**	**Choice**	**Autonomy in work methods**	**Work pace**	**Coordination mechanisms**	**Support**	**Earnings responsibility**	**Worktime**
Contract	1	−0.045	−0.075^*^	0.027	0.033	−0.009	0.311^*^	0.224^*^	0.089^*^
Econ. independency	−0.045	1	0.146^*^	0.221^*^	0.100^*^	−0.156^*^	0.084^*^	−0.050	0.164^*^
Choice	−0.075^*^	0.146^*^	1	0.110^*^	0.034	−0.030	−0.079^*^	−0.071^*^	0.124^*^
Autonomy in work methods	0.027	0.221^*^	0.110^*^	1	0.432^*^	−0.116^*^	0.077^*^	−0.050	0.291^*^
Autonomy in work Pace	0.033	0.100^*^	0.034	0.432^*^	1	−0.094^*^	0.083^*^	−0.029	0.199^*^
Coordination mechanisms	−0.009	−0.156^*^	−0.030	−0.116^*^	−0.094^*^	1	0.037	0.000	−0.008
Support	0.311^*^	0.084^*^	−0.079^*^	0.077^*^	0.083^*^	0.037	1	0.188^*^	0.234^*^
Earnings responsibility	0.224^*^	−0.050	−0.071^*^	−0.050	−0.029	0.000	0.188^*^	1	−0.005
Worktime	0.089^*^	0.164^*^	0.124^*^	0.291^*^	0.199^*^	−0.008	0.234^*^	−0.005	1

* *p < 0.01 (bilateral)*.

The results show us that most sub-dimensions are not correlated (*r* < 0.10 and/or *p* > 0.05) or weakly correlated (*r* < 0.30). However, we observe an important correlation between autonomy in work methods and autonomy in work pace (*r* = 0.432; *p* < 0.001). Therefore, to avoid overweighting one factor in our cluster analysis and delivering misguided results due to collinearity, we decided to merge the indicators of work pace and methods into one single new construct calculated with the mean of the two dimensions.

These preliminary results show that the various sub-dimensions of our matrix are not systematically correlated. These results support the idea that autonomy at work must be considered as a multidimensional concept as we can hardly isolate specific variables likely to predict the others. Each dimension brings its own share of new information on the autonomy at work of IPros.

### Building an Empirical Typology

#### Procedure

Building on these indicators, we looked for groups of workers sharing the same patterns of results on the various dimensions of autonomy. We used a hierarchical clustering algorithm with a consolidation of the classes using k-means algorithm. The purpose of (hierarchical) cluster analyses is not to find a classification based on identification criteria (which is the goal of a conceptual classification) but rather to group individuals according to their similarity on multiple dimensions. The hierarchical clustering algorithm groups observations according to their similarity. The latter is calculated with Euclidian distance and Ward's linkage (Attewell and Monaghan, 2015). Hierarchical clustering is a bottom-up approach to clustering. In our case, each worker is considered as a single cluster at the beginning and then is successively merged in pairs of clusters that are the most similar on the different dimensions of autonomy until all clusters have been merged into one single cluster that contains all workers. Once the expected number of clusters is reached, the k-means algorithm calculates their center and categorizes each observation according to the closest cluster center. This consolidation of the hierarchical clustering methods is associated with more robust classifications. Before applying the clustering algorithms, indicators are standardized, missing values are imputed according to the proximity between individuals and the relations between the indicators (Josse and Husson, 2016), and the relative weight of individuals (controlling for survey design, post-stratification, and supranational weights) is considered by using the weighting variable provided by Eurofound.

**Figure 6:**
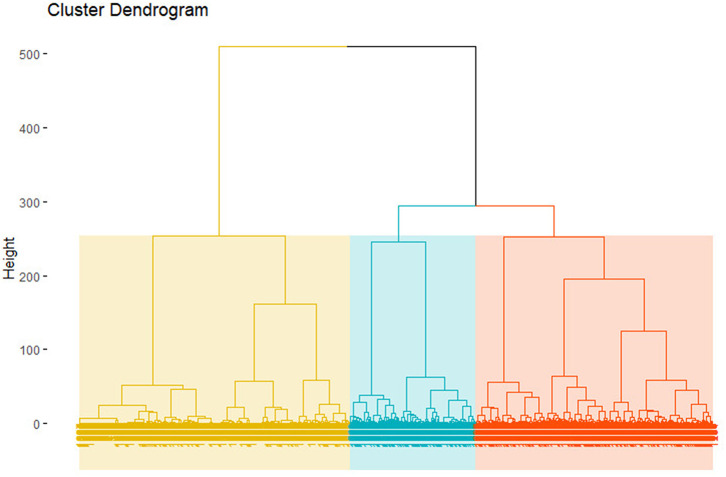
Cluster dendrogram.

To select the optimal number of clusters, we looked for a significant breakdown in the gain of internal consistency of clusters (how similar are the members of one cluster). This can be done by calculating the heterogeneity of clusters, measured with the Total Within Sum of Squares (TWSS), and looking for a breaking point in the consistency gain, according to the “elbow” method (Attewell and Monaghan, 2015). There is no significant drop in TWSS that would prescribe the use of a specific number of clusters. Therefore, we relied on the interpretability of clusters to choose the number of categories to produce. We tested solutions from 2 to 7 clusters. The results with three clusters seem to produce the most interpretable clusters. Figure 6 displays the cluster dendrogram resulting from the three clusters option.

#### Characteristics of the Clusters

It is worth noting that we first conducted our analysis with four clusters. This analysis resulted in a similar structure with two groups differing from the main group of autonomous Ipros either on their level of dependency or support. In addition to these distinctions, we also had a fourth group that distinguished itself from the autonomous Ipros by a lower score on the choice for self-employment. This however appeared not sufficient to keep this group as a separate cluster. Even though the possibility to choose the self-employed status is stressed out as an important dimension in the literature on precariousness (Kautonen et al., 2010; Leighton and McKeown, 2015), our results show that it is not necessarily related to other dimensions of autonomy: the two clusters do not differ on other dimensions than choice. However, in the subsequent analyses, this fourth cluster of involuntary Ipros showed a higher proportion of female workers, a lower education, a lower level of work satisfaction and a lower belief that their job offers good prospects for career advancement. This shows that, even though the question of choice does not necessarily correlate with the other dimensions of autonomy, it remains associated with some socio-demographic profiles and levels of job satisfaction.

**Figure 7:**
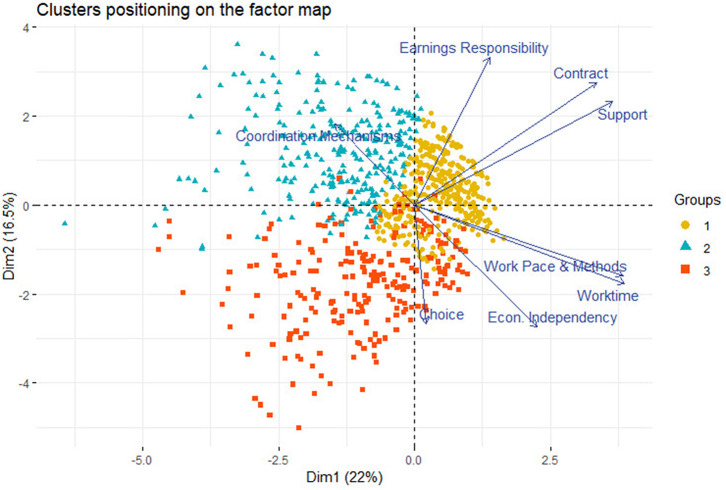
Factor map with clusters.

In a second step, the clustering analysis produced three clusters: the latter can be displayed on a factor map (Figure 7). This factor map synthetizes the information given by the eight indicators on two axes (principal components). We can observe the differences between the clusters according to their positions on the map in relation with the different indicators.

To reach a more precise understanding of our clusters, we can look at their means on each dimension of autonomy (cfr. Table 4). The difference between the means of the clusters were tested pairwise with a *t*-test. Our indicators do not always follow a normal distribution. However, the *t*-test is considered robust enough to handle non-normal distributions if the samples are large (Snijders, 2011).

**Table 4:** Clusters means.

	**Autonomous Ipros**	**Economically dependent Ipros**	**Supported Ipros**
	***N* = 791**	***N* = 284**	***N* = 270**
Contract	1.00_a_	0.99_a_	0.88_b_
Econ. independency	0.76_a_	0.39_b_	0.75_a_
Choice	0.72_a_	0.56_b_	0.81_c_
Work pace and methods	0.95_a_	0.65_b_	0.93_a_
Coordination mechanisms	0.54_a_	0.68_b_	0.57_a_
Support	0.87_a_	0.58_b_	0.53_b_
Earnings responsibility	1.00_a_	0.98_b_	0.69_c_
Worktime	0.95_a_	0.55_b_	0.87_c_

*Values in the same row and subtable not sharing the same subscript are significantly different at p < 0.05 in the two-sided test of equality for column means. Cells with no subscript are not included in the test. Tests assume equal variances. Tests are adjusted for all pairwise comparisons within a row of each innermost subtable using the Bonferroni correction*.

The first cluster is made of Ipros who are autonomous on most dimensions. They enjoy a large autonomy in terms of work status, work content and working conditions. They correspond to the standard view of self-employed workers. We labeled this group as the “Autonomous IPros.” They represent the majority of the Ipros in EU28 (59%).

The second cluster is made of more economically dependent Ipros with less autonomy regarding their work methods, pace and working time arrangements while being self-responsible for their contract arrangements and the generation of income. They are less likely to choose the self-employed status. Conversely, they enjoy higher support from colleagues and business partners with whom they must coordinate. They represent 21% of our sample. This group is called the “Economically dependent Ipros.”

The third cluster displays lower scores in terms of self-responsibility in their contractual arrangements and generation of income while enjoying high autonomy in terms of work content. Such workers receive more support from colleagues and partners. This group accounts for about 20% of the sample. These workers may be considered as the “Supported Ipros.”

#### Clusters Description Using Variables From the Survey

##### Demographics and activity

Some demographic variables and indicators of economic activity can be associated with each cluster (cfr. Table 5). Compared to the two other clusters, women are slightly underrepresented in the supported Ipros. In terms of education, the supported Ipros seems to have a lower proportion of lower-educated workers. Autonomous Ipros are concentrated in “other” service activities (32%) while economically dependent Ipros are more present in health and social work sectors (22%). Supported Ipros are prevailing in professional, scientific and technical activities (30%).

**Table 5:** Demographic variables and economic activity in the different clusters.

**Clusters**		**Autonomous Ipros (%)**	**Economically dependent Ipros (%)**	**Supported Ipros (%)**
Gender	Male	43.0	41.9	51.1
	Female	57.0	58.1	48.9
Second job	No other paid job	90.9	86.9	87.4
	Regular second job	3.9	6.0	7.0
	Occasional second job	4.4	6.7	5.6
	Other	0.8	0.4	0.0
Education	Lower secondary education or lower	9.6	16.6	5.6
	Upper secondary education	30.7	27.9	30.1
	Short post-secondary education	19.7	17.7	24.5
	Bachelor or higher	39.9	37.8	39.8
Economic activity (NACE)	J Information and communication	6.8	13.3	11.0
	K Financial and insurance activities	4.2	3.0	9.8
	L Real estate activities	2.7	4.3	6.5
	M Professional, scientific and technical activities	22.8	15.9	30.2
	N Administrative and support service activities	0.0	0.0	0.0
	P Education	8.1	11.2	3.3
	Q Human health and social work activities	15.4	21.9	11.8
	R Arts, entertainment and recreation	8.1	12.0	9.0
	S Other service activities	31.8	18.5	18.4

##### Independence

Table 6 displays the results of the clusters on two questions used by Eurofound to evaluate the dependency of self-employed workers. As expected, economically dependent Ipros have less authority than their counterparts regarding the possibility to hire or dismiss employees. Indeed, even though we focus on self-employed workers without employees, not having the authority to hire an employee if required is an indicator of dependency or some sort of subordination according to Eurofound (2013). They, and the supported Ipros, are also more likely to be paid an agreed fee on a weekly or monthly basis, which is closer to a subordinated employment relationship.

**Table 6:** Independence variables in the different clusters.

**Clusters**		**Autonomous Ipros (%)**	**Economically dependent Ipros (%)**	**Supported Ipros (%)**
Q9a—have the authority to dismiss or hire employees	Yes	65.1	36.5	68.0
	No	34.9	63.5	32.0
Q9b—Get paid an agreed fee on a weekly or a monthly basis	Yes	33.0	52.5	48.1
	No	67.0	47.5	51.9

##### Self-employment situation

Table 7 provides data on multiple questions regarding the subjective appreciations of the self-employment situation. Supported Ipros have a higher proportion (40%) of workers who consider themselves as financially safe in case of a long-term sickness. Supported Ipros and Autonomous Ipros are in vast majority enjoying being their own boss. This tendency is still present but less pronounced for the economically dependent Ipros. Seven percent of the economically dependent Ipros say they dislike being their own boss while this proportion does not go above 1.5% in the two other clusters. More than half of the Supported Ipros and Autonomous Ipros consider it is easy to find new customers. Around 1 out of 5 workers in these clusters find it hard while, for the economically dependent, 1 out of 3 workers find it difficult. While the vast majority of the Autonomous Ipros (79%) and Supported Ipros (65%) strongly agree with the statement that they are making the most important decisions about how the business is run, this proportion drops at only 44% for the economically dependent Ipros. Moreover, 15% of the workers from this cluster disagree with this statement while it is never more than 3% for the two other clusters.

**Table 7:** Self-employment situation variables in the different clusters.

**Clusters**		**Autonomous Ipros (%)**	**Economically dependent Ipros (%)**	**Supported Ipros (%)**
Q91a—if I had a long-term sickness, I would be financially secure	Strongly agree	11.9	14.0	19.5
	Tend to agree	16.8	12.1	20.7
	Neither agree nor disagree	13.9	15.8	17.3
	Tend to disagree	21.7	21.9	19.5
	Strongly disagree	35.7	36.2	22.9
Q91b—I enjoy being my own boss	Strongly agree	75.4	51.3	81.0
	Tend to agree	18.5	26.0	14.5
	Neither agree nor disagree	5.2	15.8	3.0
	Tend to disagree	0.5	3.8	0.7
	Strongly disagree	0.4	3.0	0.7
Q91c—It is easy for me to find new customers	Strongly agree	17.0	16.1	16.7
	Tend to agree	34.4	22.2	34.9
	Neither agree nor disagree	28.6	27.8	27.5
	Tend to disagree	13.1	19.1	14.3
	Strongly disagree	6.9	14.8	6.6
Q91d—I find it hard for me bearing the responsibility for running my business	Strongly agree	6.2	10.9	5.3
	Tend to agree	14.8	15.5	15.9
	Neither agree nor disagree	16.7	19.7	18.9
	Tend to disagree	21.7	25.6	21.6
	Strongly disagree	40.6	28.2	38.3
Q91e—I make the most important decisions on how the business is run	Strongly agree	79.4	44.7	65.5
	Tend to agree	15.8	23.0	22.5
	Neither agree nor disagree	3.5	16.8	8.2
	Tend to disagree	0.8	9.4	1.5
	Strongly disagree	0.5	6.1	2.2

##### Job satisfaction and prospects

Table 8 shows that most IPros are satisfied with their working conditions. Almost half of the Supported Ipros and more than 40% of the Autonomous Ipros declare being very satisfied with their working conditions while this proportion remains under 30% for the economically dependent Ipros. More than half of the Autonomous and Supported Ipros also believes their job offers good prospects for career advancement. While it is the case for <40% of the economically dependent Ipros.

**Table 8:** Job satisfaction and prospects in the different clusters.

**Clusters**		**Autonomous Ipros (%)**	**Economically dependent Ipros (%)**	**Supported Ipros (%)**
Q88—Satisfaction with working conditions	Very satisfied	41.8	29.9	48.5
	Satisfied	48.8	57.4	46.3
	Not very satisfied	7.7	10.6	5.2
	Not at all satisfied	1.6	2.1	0.0
Q89b—My job offers good prospects for career advancement	Strongly agree	21.2	14.6	37.0
	Tend to agree	29.1	22.3	27.8
	Neither agree nor disagree	25.3	22.7	16.3
	Tend to disagree	11.2	18.0	10.1
	Strongly disagree	13.3	22.3	8.8

## Discussion

While most IPros enjoy high levels of autonomy on the different dimensions of our grid, our univariate analysis of indicators also pointed that there is a non-negligible part of this population with lower scores on some dimensions. These lower scores entail negative situations already pointed out in the literature, such as being pushed toward self-employment (Fleming, 2017), being economically dependent (de Peuter, 2011; Standing, 2011; Bergvall-Kåreborn and Howcroft, 2013), having strict guidelines to follow or not being responsible for working time arrangements. But lower scores on some dimensions might have a positive impact on the work quality. A low score on the support dimension means that the worker enjoys less autonomy and accesses shared expertise and support from managers, colleagues, and/or teammates. Our approach therefore provides a more comprehensive vision of autonomy at work of IPros by using multiple dimensions on the same data.

This approach leads us better understand risks and opportunities associated with the work of Ipros. Workers with high levels of autonomy (the majority of Ipros) may indeed face the following risks: no (or discontinuous) access to social protection, low access to shared expertise and support, self-responsibility for skills development and for generating a steady income flow, etc. On the other side, high autonomy may also offer benefits in terms of freedom of choice for the job status, broader guidelines allowing job crafting, self-responsibility for workload, and work pace, self-responsibility for space, and time arrangements, etc.

However, Ipros may obtain lower scores on some dimensions of autonomy, which leads them face some risks such as: higher economic dependency, forced orientation to casual work, strict guidelines reducing the possibilities of job crafting, less responsibility over workload, and work pace, etc. There are however some benefits associated with low levels of autonomy. If most of them remain inaccessible to the majority of Ipros due to their self-employed status (secure legal status, continuous access to social protection), our results showed that a minority of these workers may enjoy support from their colleagues and managers.

Autonomous Ipros may be considered as autonomous on every dimension. They are their own boss, make the most important decisions about how their business is run, enjoy great levels of responsibility for their work content and working conditions and are relatively satisfied.

Individual situations of economically dependent Ipros are blended with high autonomy on most dimensions and lower scores on some dimensions. They are more likely to be dependent on one single business partner and, while this might bring advantages in terms of organizational support, they do not enjoy the same levels of autonomy as other Ipros when considering work content and working conditions: they are associated with lower job satisfaction scores and more precarious self-employment situations. This could result from purely transactional arrangements with client organizations. In this perspective, the use of contract work is just a question of business optimization, via cost reduction and/or flexible responses to market variations. Client organizations are not led to invest such short-term business relationships: work arrangements are mainly focused on performances and compliance with the terms and conditions of contracts, with low consideration on the development of human capital. This “low road strategy” (Gautié and Schmitt, 2010) is very frequent in mass-market industries.

Conversely, the positive scores obtained in the supported Ipros cluster probably originate from another attitude of client organizations: more emphasis is then put on skills development, individual commitment, self-determination rather than compliance with command-and-control systems, intensive communication and participation. Indeed, some organizations tend to develop such a “high road strategy” (Gautié and Schmitt, 2010) with Ipros, in order to build a genuine partnership with them due to the uniqueness of their human capital (Lepak and Snell, 1999). In line with previous research (Koene and van Riemsdijk, 2005; Coyle-Shapiro et al., 2006), a survey among 375 Ipros working in a large range of Australian organizations (McKeown and Cochrane, 2017) showed that organizational support—offered either by client organizations or labor market intermediaries—significantly predicts their affective commitment, which reinforces their potential contribution to organizational performances. Workers from Supported Ipros, who enjoy higher levels of autonomy on work content and working conditions while benefiting from more organizational support are also amongst the most satisfied with their working conditions and their self-employment situation.

## Conclusive Remarks

Therefore, the future of career management might be based on the ability of HR managers to grasp the various and changing ways through which Ipros look for and enact autonomy at work, in order to provide them with appropriate answers to the growing risks they experience in terms of access to social protection, forced orientation to the self-employment status, economic dependence on one single client, limited possibilities of job crafting, limited support to shared professional expertise, limited possibilities of skills development, discontinuity of incomes, etc. The choice of this “high road strategy” (Osterman, 2018), involving external workers in a more inclusive perspective, is not only based on “moral” considerations on what should be done in order to improve the job quality of Ipros. More and more HR managers become aware of the growing risks they may face when their company is using self-employment arrangements. Disloyal and opportunistic behaviors, lack of visibility on contractors, emergence of new labor market intermediaries, and quasi unions (Hirsch and Seiner, 2018), potential degradation of the service quality, negative signals sent to regular employees leading to disengagement (von Hippel and Kalokerinos, 2012), lack of collective learning and exchange (Grugulis and Stoyanova, 2011), loss of expertise and innovation, etc. are increasingly considered and lead to the development of “total workforce management” initiatives. A growing body of literature advocates for a better management of such a hybrid workforce (Cascio and Boudreau, 2017). Even if the dominant approach so far looks like a new rhetoric, mostly developed by consultants and HR technology vendors, it paves the way to a new role likely to be played by HR managers.

Younger and Smallwood (2016) point out that companies that consider external workers with the same attention as permanent workers get the highest commitment from this flexible workforce. The same argument was already highlighted in a study on temporary workers by Koene and van Riemsdijk (2005). Multiple empirical studies (Kuhn and Maleki, 2017; McKeown and Cochrane, 2017) argue that tailored initiatives including external workers (high road strategy) give modern organizations significant competitive advantages compared to those neglecting the contributions of external workers. In order to do so, HR managers have to learn new cooperation games, not only with internal actors (purchase, line and project managers, as suggested by Keegan et al., 2012) but also with their counterparts in other client organizations and with emerging third-party actors such as labor market intermediaries (Bonet et al., 2013; Lorquet et al., 2018) and quasi unions voicing the concerns of self-employed and freelance workers (Hirsch and Seiner, 2018).

We must keep in mind some limitations of this research while looking at its findings. First, our empirical test was based on a secondary analysis of existing data (EWCS). We were thus unable to find relevant information for each component of our conceptual framework. Further empirical investigations will be needed in order to gather more relevant primary data according to our analytical grid. Second, the use of cross-sectional data makes it impossible to look at the evolution of self-employment arrangements over time. The exploratory character of our clustering methods gives us insights about associations between variables grouped in each cluster and other descriptive variables but these methods prevent us from identifying clear causal patterns. In line with our methodological choices, our argument is not positivist. We do not pretend to find objective existing categories of workers but to shed light on the variety of experiences of autonomy and the perception of risks associated with them. We also tried to use factual indicators in the construction of clusters. More subjective questions about contractual arrangements and job satisfaction are needed to better understand the concrete experiences of autonomy at work: some of them were used as illustrative variables to better highlight the differences between clusters.

Still, our results represent an important contribution to the literature on new forms of employment. Our findings bring a nuanced take on the binary considerations on autonomy at work of independent professionals, either presented as highly autonomous workers benefitting from flexible work arrangements or, conversely, associated with precarious work arrangements and painful working conditions. Our findings show the added value of an empirical typology that helps better understand the experience of autonomy in non-standard work arrangements and paves the way to the development of more appropriate policies, taking account of the diversity of IPros' working situations. It should be further validated on other datasets in order to identify relevant links between the employment arrangements for IPros and other variables such as the well-being or job quality.

## Data Availability Statement

The datasets generated for this study are available on request to the corresponding author.

## Author Contributions

FP has provided theoretical support, reviewing, and editing of the paper. LF was responsible for the data acquisition and analysis and drafting of the paper. All authors are responsible for the study design and methodology.

## Conflict of Interest

The authors declare that the research was conducted in the absence of any commercial or financial relationships that could be construed as a potential conflict of interest.

## Footnotes

^1^Statistical classification of economic activities in the European community.

^2^For the sake of brevity, we do not develop the calculation of each indicator in this paper. Would you be interested in this process, please contact the authors for a methodological Annex (in Supplementary Material).

^3^The classes relate to the original questions used for computing indicators shown in Table 2.

^4^Details of the distribution for each indicator are available in the methodological Annex (Supplementary Material).
