# Supplementary material for: Emerging Forms of Precariousness Related to Autonomy at Work: Toward an Empirical Typology
Source: Front Sociol. 2020 May 22;5:34. doi: 10.3389/fsoc.2020.00034 (PMC8022474; doi:10.3389/fsoc.2020.00034)
Supplement: Supplementary file 1 [file Table_1.DOCX]

**Emerging forms of precariousness related to autonomy at work: Proposals for a statistical measurement**

**Methodological annex**

**Louis Florin, LENTIC, HEC-ULiège**

Following the work of Pichault and Mckeown (2019), we decided to study autonomy as a central feature of new employment arrangements.

In this methodological annex, we present in detail the construction and distribution of each indicator based on their conceptual grid of autonomy.

As each subdimension of autonomy has a different number of key variables (survey questions giving us information about this particular dimension), we must calculate a synthetic indicator for each dimension^[[1]](#footnote-1)^. These indicators are normalized on a scale from 0 to 1. A score of 0 represents the lowest autonomy and a score of 1 represents the highest autonomy.

1. **Work Status**
   1. **Autonomy in the contractual arrangements**

We used the question 8b to determine the level of autonomy in the contractual arrangements for the self-employed workers:

Q8b Select the category or categories which apply to your main paid job?

[MULTIPLE ANSWERS] 0 or 1

1. Sole director of own business

2. A partner in a business or professional practice

3. Working for yourself

4. Working as a sub-contractor

5. Doing freelance work

6. Paid a salary or a wage by an agency

We distributed the scores on a from 0 to 1 according to the different possibilities in the conceptual grid of autonomy regarding contractual arrangements (Independent contractor, supported independent contractor, temporary worker, regular employee). Logically, as we work with self-employed workers in this paper, we have no answer that fits in the regular employee category. The scores were attributed as follows:

| Question | Independent contractor | Supported independent contractor | Temporary worker | Regular employee |
| --- | --- | --- | --- | --- |
| Q8b | 1,3,5 | 2,4 | 6 |  |
| **Score** | **1** | **0.66** | **0.33** | **0** |

If the respondent gave multiple answers, we computed the mean of his scores^[[2]](#footnote-2)^. This calculation gives the following results:


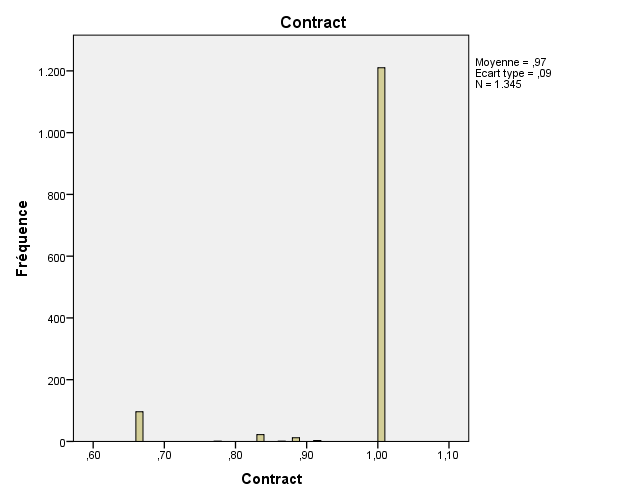


|  | Contract |
| --- | --- |
| Minimum | 0,66 |
| Maximum | 1 |
| Mean | 0,9715 |
| Standard deviation | 0,08999 |

- 1. **Insurance and social rights**

Regarding this dimension, we could only identify question 91a:

Q91a - A. If I had a long-term sickness, I would be financially secure? (Strongly agree, Tend to agree, Neither agree nor disagree, Tend to disagree, Strongly disagree)

However, this question only informs us about the capacity of the worker to face a period of insecurity, but it lacks information about the source of potential security (does it come from private insurance? support from an intermediary? etc.).

Therefore, we did not compute an indicator for this dimension, but we use question 91a among other dependent variables to describe the clusters.

- 1. **Economic dependency**

For the self-employed workers, economic independency can be studied with two questions :

Q9d Regarding your business, do you generally, have more than one client or customer?

1=Yes / 2=No

Q102 What proportion of revenue do you receive from your most important client?

1=Less than 50%

2=50 to 75%

3=More than 75%

We defined a score of 0 for economic independency when the worker has only one client/customer. We attributed a score of 1 when the worker perceives less than 50% of his revenue from one client. The two other responses were distributed on the continuum as follows:

| Question | <50% of revenue from main client | >50% and <75% of revenue from main client | >75% of revenue from main client | 1 client |
| --- | --- | --- | --- | --- |
| Q9d |  |  |  | 2 |
| Q11 | 1 | 2 | 3 |  |
| **Score** | **1** | **0.66** | **0.33** | **0** |


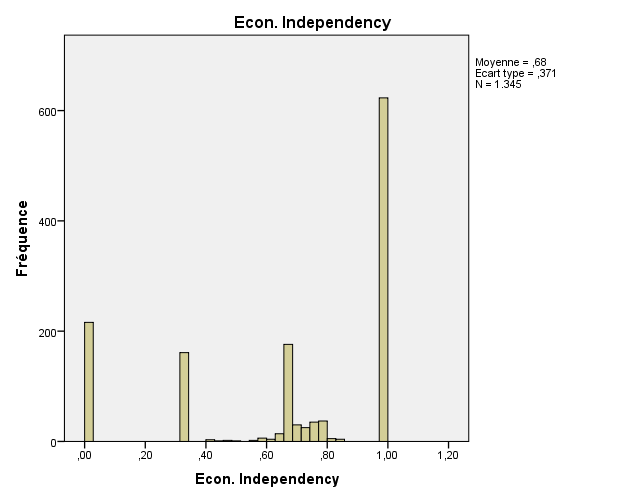
To compute the indicator, we chose to take the minimum score of the two questions. For example, if a worker declares perceiving less than 50% of his revenue from a single client but still declares having only one client (unlikely situation), we still attributed him a score of 0. This is because computing the mean of the two questions would make no sense here and because we want to identify the cases of economic dependency.

|  | Econ. Independency |
| --- | --- |
| Minimum | 0 |
| Maximum | 1 |
| Mean | 0,6794 |
| Standard deviation | 0,37083 |

- 1. **Choice for self-employed work**

The question of autonomy in the choice for self-employed work is asked with Q10:

Q10 When you became self-employed, was it mainly through your own personal preference or

because you had no other alternatives for work?

1. Mainly through own personal preferences

2. No other alternatives for work

3. A combination of both (spontaneous)

4. Neither of these reasons (spontaneous)

For this indicator, we simply computed the indicator by attributing scores as follows:

| Question | Personal choice |  | Constrained choice |
| --- | --- | --- | --- |
| Q10 | 1 | 3,4 | 2 |
| **Score** | **1** | **0.5** | **0** |


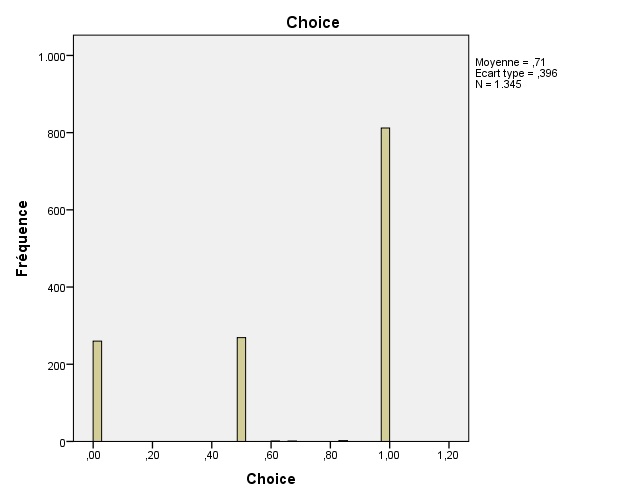
This gives the following results:

|  | Choice |
| --- | --- |
| Minimum | 0 |
| Maximum | 1 |
| Mean | 0,7059 |
| Standard deviation | 0,39645 |

1. **Work content**
   1. **Autonomy in work methods**

The dimension of autonomy in work methods can be covered with questions q54b, Q61i and q61n.

Q54b Are you able to choose or change your methods of work?

1. Yes
2. No

Q61I. You are able to apply your own ideas in your work

Q61N. You can influence decisions that are important for your work

1. Always
2. Most of the time
3. Sometimes
4. Rarely
5. Never

To compute our indicator, we compute a new variable for each question and attributes scores between 0 and 1 for each of them. For Q54b, response 1 indicates greater autonomy than response 2. For questions 61i and 61n, we distributed the scores on a continuum from 0 to 1 as follows:

| Question | Autonomy in work methods | | | | | |
| --- | --- | --- | --- | --- | --- | --- |
|  | **Broad guidelines allowing job crafting** | | | **Precise specifications preventing job crafting** | | |
| **Q54** | 1 | / | / | | / | 2 |
| **Q61i and Q61n** | 1 | 2 | 3 | | 4 | 5 |
| **Score attr** | **1** | **0.75** | **0.5** | | **0.25** | **0** |


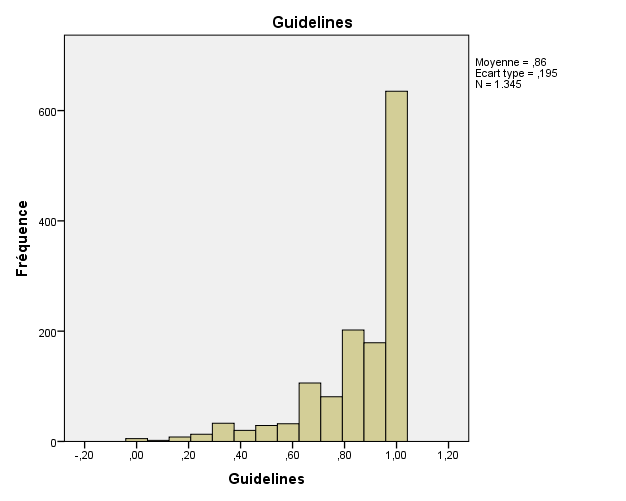
We computed the indicator by calculating the mean of each of the recoded variables. This gives the following results:

|  | Work methods |
| --- | --- |
| Minimum | 0 |
| Maximum | 1 |
| Mean | 0,8595 |
| Standard deviation | 0,19477 |

- 1. **Autonomy in work pace**

Information regarding the autonomy in the work pace is asked in question 54c:

Q54cAre you able to choose or change your speed or rate of work?

1=Yes

2=No

This question only let us compute two possible scores for this indicator:

| Question | Autonomy in the work pace | |
| --- | --- | --- |
|  | **Workpace, workload at own discretion** | **Workpace, workload imposed by clients** |
| **Q54c** | 1 | 2 |
| **Score** | **1** | **0** |

This gives us the following results:


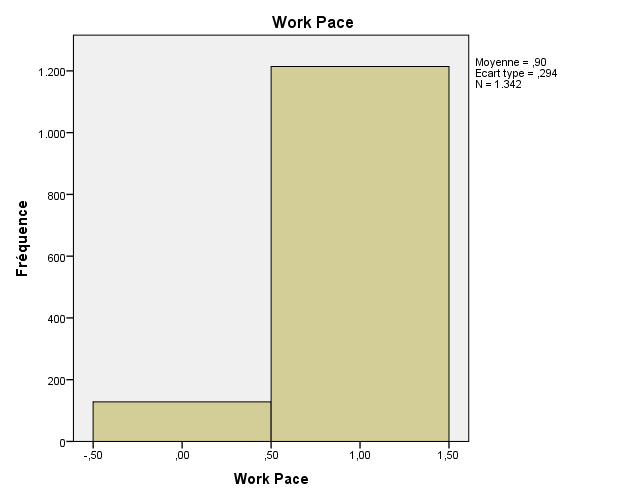


|  | Work Pace |
| --- | --- |
| Minimum | 0 |
| Maximum | 1 |
| Mean | 0,9046 |
| Standard deviation | 0,29385 |

- 1. **Coordination mechanisms**

We can find information about the work coordination in question 50:

Q50 On the whole, is your pace of work dependent on...

A. The work done by colleagues

B. Direct demands from people such as customers,

passengers, pupils, patients, etc.

C. Numerical production targets or performance targets

D. Automatic speed of a machine or movement of a product

E. The direct control of your boss

1=Yes

2=No

For the coordination mechanisms, Pichault & Mckeown suggest the following: “According to Mintzberg’s typology of coordination mechanisms (1979, 1983), hierarchical mechanisms imposed by clients (direct supervision, standardisation of work processes) reduce the autonomy of independent professionals, as is usually the case with crowd workers (De Stefano, 2016). Conversely, clans (mutual adjustment and standardisation of norms) give them a larger room for manoeuvre, while markets (standardisation of outcomes) can be located midway between these two poles given that independent professionals are not supervised when doing their job but have to report the results of their activities.” (2019, p.65).

Therefore, we can classify the answers of question 50 in three categories as follows:

| Question | Clans:  Mutual adjustement, Standardization of norms | Market:  Standardization of outcomes | Hierarchy:  Standardization of processes; Direct supervision |
| --- | --- | --- | --- |
| Q50a | 1 |  |  |
| Q50b |  | 1 |  |
| Q50c |  | 1 |  |
| Q50d |  |  | 1 |
| Q50e |  |  | 1 |
| Score | 0.75 | 0.5 | 0.25 |

As we have two answers linked to market coordination and hierarchy as opposed to only one answer for clan coordination, we first computed a single indicator for each of the three categories to avoid overweighting market and hierarchy in the calculation of the final indicator. The final indicator is calculated to give either the score of the single category the worker has mentioned (Clans, Market or Hierarchy) or to compute the mean of two scores if the worker has mentioned multiple category (for example if he answers that his work pace is dependent on the work done by colleagues (Q50a – mutual adjustment/clan coordination) and direct demands from clients (Q50b – standardization of outcomes/market coordination), he would get as a score the mean of 0.75 (Q50a) and 0.5 (Q50b) which will be 0.625. This calculation gives the following results:

|  | Coordination Mechanisms |
| --- | --- |
| Minimum | 0,25 |
| Maximum | 1 |
| Mean | 0,5756 |
| Standard deviation | 0,19092 |


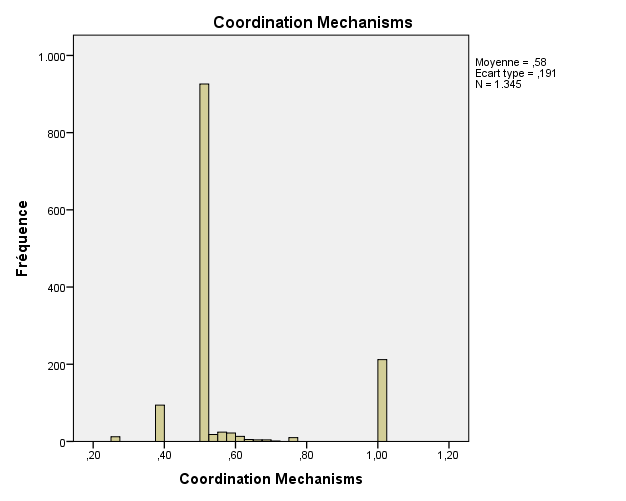


- 1. **Support/Access to shared expertise**

The EWCS survey provides information about organizational support through two questions. One question on teamwork (q58) and one question on colleagues’ support (q61a):

Q58 Travaillez-vous dans un groupe ou une équipe qui a des tâches communes et qui peut planifier son travail ?

1=Oui

2=Non

Q61A. Your colleagues help and support you

1. Always

2. Most of the time

3. Sometimes

4. Rarely

5. Never

As our goal is to create indicators of autonomy, for this indicator of support/access to shared expertise, less support would represent greater autonomy and therefore a score of 1. On the contrary, more support would mean less autonomy and therefore a lower score.

For question 58, people working in teams enjoy greater access to shared expertise and support, meaning a lower score on autonomy than people working alone. For question 61a, the scale goes from a greater support (less autonomy) to the absence of support from colleagues (more autonomy). We distributed the scores on a continuum from 0 to 1 as follows:

| Question | Support/access to shared expertise | | | | | |
| --- | --- | --- | --- | --- | --- | --- |
|  | **Strong support and access to shared expertise** | | | **Low support and access to shared expertise** | | |
| **Q58** | 1 | / | / | | / | 2 |
| **Q61a et Q61b** | 1 | 2 | 3 | | 4 | 5 |
| **Score** | **0** | **0.25** | **0.5** | | **0.75** | **1** |


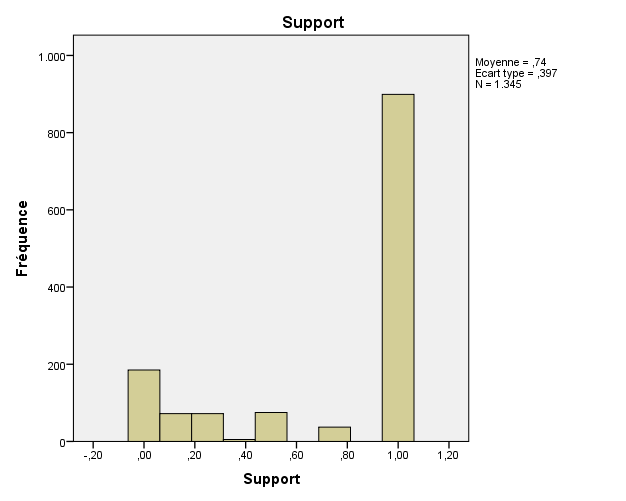
This calculation gives the following results:

|  | Support |
| --- | --- |
| Minimum | 0 |
| Maximum | 1 |
| Mean | 0,7384 |
| Standard deviation | 0,39677 |

1. **Working conditions**
   1. **Responsibility for skills development**

The questions in the 2015 EWCS did not provide us with relevant information about this dimension. There are some questions dedicated to skills development and training (65,66,67,68 and 69) but they do not give information about the responsibility and autonomy on this matter.

- 1. **Responsibility for generating income**

For self-employed workers, question 103 gives information about the types of revenue the workers get:

Q103. What do your earnings from your main business include?

A. Income from self-employment such as own business, profession or farm.

B. Payments based on the overall performance of the company (profit sharing scheme) or partnership where you work

C. Income from shares in the company you work for

1 = Yes

2 = No

In the survey, we did not get information about the multiple possibilities identified by Pichault & Mckeown (2019) such as intervention of intermediaries, the negotiated or standardized character of the earnings but we still classified these answers according to the level of autonomy and responsibility placed on the worker. The scores were attributed as follows:

| Question | Based on the performance of the company | Direct income from own business |
| --- | --- | --- |
| Q103a |  | 1 |
| Q103b | 1 |  |
| Q103c | 1 |  |
| Score | 0,33 | 1 |


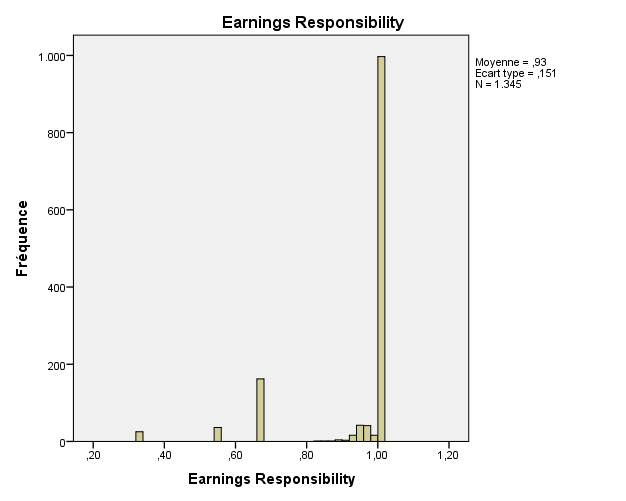
As the respondent can give multiple answers, we computed the mean of the scores to get the final indicator. This calculation gives the following results:

|  | Earnings Responsibility |
| --- | --- |
| Minimum | 0,33 |
| Maximum | 1 |
| Mean | 0,9309 |
| Standard deviation | 0,15071 |

- 1. **Self-responsibility for time and space arrangements**

For this dimension, we only have relevant information about the autonomy in the worktime arrangements. Indeed, as with other dimensions, we lack information about the responsibility and autonomy for workplace arrangements. Q42 provides us with information on the autonomy in the worktime arrangements:

Q42 How are your working time arrangements set?

1=They are set by the company / organisation with no possibility for changes

2=You can choose between several fixed working schedules determined by the company/organisation

3=You can adapt your working hours within certain limits (e.g. flextime)

4=Your working hours are entirely determined by yourself

We attributed a score for each possible answer, from 0 for the predetermined worktime set by the organization to 1 for complete autonomy. The scores are attributed as follows:

| Question | Set by the company | Choice between several working schedules determined by the company | Adaptable working hours within certain limits | Complete autonomy |
| --- | --- | --- | --- | --- |
| Q42 | 1 | 2 | 3 | 4 |
| **Score** | **0** | **0.33** | **0.66** | **1** |

After computing the indicator by using the scores mentioned above, we obtain the following results:


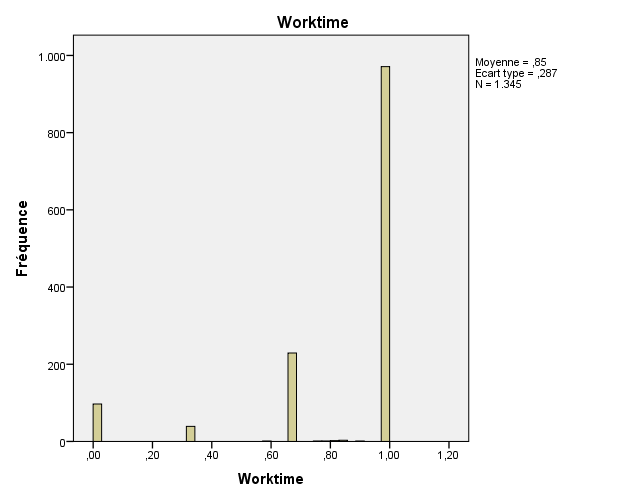


|  | Worktime |
| --- | --- |
| Minimum | 0 |
| Maximum | 1 |
| Mean | 0,8493 |
| Standard deviation | 0,28714 |

References:

Pichault, F. and Mckeown, T. (2019). Autonomy at work in the gig economy: analysing work status, work content and working conditions of independent professionals. New Technology, Work and Employment 34(1)

1. This annex explains the calculation methods for each dimension. Would you be further interested in using these indicators, you can contact the author to get the SPSS syntax file to apply on the EWCS survey results. [↑](#footnote-ref-1)
2. For example, someone that would have answered 1 – sole director of my own business – and 4 – working as a subcontractor – would be given the score of (1+0.66)/2 = 0.83). [↑](#footnote-ref-2)
